# Optimal cardiopulmonary resuscitation duration for favorable neurological outcomes after out-of-hospital cardiac arrest

**DOI:** 10.1186/s13049-022-00993-8

**Published:** 2022-01-15

**Authors:** SungJoon Park, Sung Woo Lee, Kap Su Han, Eui Jung Lee, Dong-Hyun Jang, Si Jin Lee, Ji Sung Lee, Su Jin Kim, for the Korean Cardiac Arrest Research Consortium (KoCARC) Investigators

**Affiliations:** 1 Department of Emergency Medicine, College of Medicine, Korea University, Inchon-ro 73, Seongbuk-gu, Seoul, 02841 Republic of Korea; 2 Clinical Research Center, Asan Medical Center, 88 Olympic-ro 43-gil, songpa-gu, Seoul, 05505 Korea

**Keywords:** Cardiac arrest, Cardiopulmonary resuscitation, Neurological outcome, Out-of-hospital cardiac arrest, Resuscitation

## Abstract

**Background:**

A favorable neurological outcome is closely related to patient characteristics and total cardiopulmonary resuscitation (CPR) duration. The total CPR duration consists of pre-hospital and in-hospital durations. To date, consensus is lacking on the optimal total CPR duration. Therefore, this study aimed to determine the upper limit of total CPR duration, the optimal cut-off time at the pre-hospital level, and the time to switch from conventional CPR to alternative CPR such as extracorporeal CPR.

**Methods:**

We conducted a retrospective observational study using prospective, multi-center registry of out-of-hospital cardiac arrest (OHCA) patients between October 2015 and June 2019. Emergency medical service–assessed adult patients (aged ≥ 18 years) with non-traumatic OHCA were included. The primary endpoint was a favorable neurological outcome at hospital discharge.

**Results:**

Among 7914 patients with OHCA, 577 had favorable neurological outcomes. The optimal cut-off for pre-hospital CPR duration in patients with OHCA was 12 min regardless of the initial rhythm. The optimal cut-offs for total CPR duration that transitioned from conventional CPR to an alternative CPR method were 25 and 21 min in patients with initial shockable and non-shockable rhythms, respectively. In the two groups, the upper limits of total CPR duration for achieving a probability of favorable neurological outcomes < 1% were 55–62 and 24–34 min, respectively, while those for a cumulative proportion of favorable neurological outcome > 99% were 43–53 and 45–71 min, respectively.

**Conclusions:**

Herein, we identified the optimal cut-off time for transitioning from pre-hospital to in-hospital settings and from conventional CPR to alternative resuscitation. Although there is an upper limit of CPR duration, favorable neurological outcomes can be expected according to each patient’s resuscitation-related factors, despite prolonged CPR duration.

**Supplementary Information:**

The online version contains supplementary material available at 10.1186/s13049-022-00993-8.

## Background

Longer cardiopulmonary resuscitation (CPR) duration in cases of out-of-hospital cardiac arrest (OHCA) is associated with reduced favorable neurological outcomes. Moreover, consensus is lacking regarding the optimal CPR duration before termination. Therefore, determining the upper limit of CPR duration is essential for stopping futile resuscitation efforts. Although several studies have investigated the CPR duration beyond which resuscitation may be futile, they did not include cases of in-hospital CPR duration and were based on pre-hospital CPR duration [1–3].

There are regional variations in emergency medical service (EMS) systems and the role of EMS providers, such as pre-hospital interventions and termination of resuscitation (TOR) [3–5]. Even if appropriate advanced life support is implemented, identifying and correcting all the causes of OHCA in the field is challenging.

Some studies showed that extracorporeal CPR (ECPR) for refractory cardiac arrest can obtain favorable neurological outcomes [6–9]. Moreover, in one study, insufficient short duration of CPR less than 20 min in the emergency department (ED) deceased survival to discharge [10]. These results give us the idea that we need sufficient time in-hospital CPR. Also considering ECPR is primarily provided in hospital settings, more likely to have sufficient CPR duration in hospital and prepare ECPR is necessary. Therefore, the allocation of adequate overall time for pre-hospital and in-hospital CPR is necessary.

In patients with OHCA, outcomes are associated with various factors, including age, comorbidities, initial cardiac rhythm, witnessed arrest, provision of bystander CPR, and resuscitation duration [11–14]. The optimal CPR duration to achieve a favorable neurological outcome is influenced by factors that determine resuscitation outcomes [1, 2, 15, 16]. Patients who achieve pre-hospital return of spontaneous circulation (ROSC) have better outcomes than those who do not [1, 17]. Most patients with OHCA do not achieve pre-hospital ROSC prior to hospital arrival, especially in regions where TOR is not allowed in the field.

This study aimed to identify the major factors of and determine the upper limit of total CPR duration and optimal cut-off for pre- or in-hospital total CPR duration to achieve favorable neurological outcomes.

## Methods

### Data source and data collection

The Korean Cardiac Arrest Research Consortium (KoCARC), a multicenter collaborative research network of 55 participating institutions, was developed to understand various studies in the resuscitation field of patients with OHCA and strengthen cooperative efforts. The KoCARC registry includes patients with OHCA of medical etiology transported to a participating ED with resuscitation efforts. The registry excludes patients with OHCA with a documented terminal illness, those under hospice care, those currently pregnant, and those with a previously documented “Do Not Resuscitate” card [18]. After 2018, advanced directives (AD) are able to guarantee the patient’s self-determination. When an AD is completed, it is registered with the National Agency for Management of Life-sustaining Treatment, and a registration card is issued [19]. This registration card is recognized as a “Do Not Resuscitate” card because it includes the rejection of CPR. Patients with OHCA of definite non-medical etiology, including trauma, drowning, poisoning, burn, asphyxia, or hanging, were also excluded [18]. Starting in October 2015, data were collected from EMS and hospital medical records via a standardized registry form and entered into a web-based electronic database registry according to Utstein style [18]. A quality management committee comprising emergency physicians, statisticians, local research coordinators, and investigators in each ED regularly monitored and reviewed the data quality [18].

### Study design and setting

This retrospective observational study used a prospective, multi-center registry of OHCA patients between October 2015 and June 2019 from KoCARC. Patients aged < 18 years, for whom information or medical record data were missing, who were transferred from other hospitals, or who received ECPR were excluded. We extracted all data including baseline characteristics, pre-hospital environmental factors, EMS characteristics, laboratory data, in-hospital therapeutic interventions, CPR duration, and clinical outcomes from the KoCARC registry.

Pre-hospital CPR duration was defined as the time from the initiation of CPR to the achievement of sustained pre-hospital ROSC or hospital arrival. In-hospital CPR duration was defined as the time from the initiation of CPR at the hospital to the achievement of sustained ROSC or discontinuation of CPR. Total CPR duration was defined as the time from CPR initiation by EMS to the achievement of sustained ROSC or discontinuation of resuscitation.

The primary endpoint was a favorable neurological outcome at hospital discharge as determined by the cerebral performance category (CPC) score [20]. CPC scores of 1–2 were considered favorable, whereas those of 3–5 were considered poor neurological outcomes.

### Korean EMS system

In the Republic of Korea, the EMS is a centralized governmental service provided by 16 provincial headquarters of the National Fire Department covering the entire nation [21]. As of 2019, the total surface area of Korea is 100,401.3 km^2^, with a population of 51,927,000. There are 12,033 paramedics and 1474 ambulances nationwide [22]. In Korea, there is no special team of doctors, and emergency medical technicians (EMTs) and nurses are in charge of dispatch. There are standard guidelines for on-site first aid from CPR in the field to hospital transportation. According to this guideline, an automated chest compression device can be used if one paramedic has to be in charge of CPR during the transfer. Additionally, the use of metronomes and chest compression quality measuring devices are recommended to monitor chest compression [23]. Two or three emergency paramedics work together, including at least one level 1 EMT who can administer intravenous fluids, perform endotracheal intubation, and apply an automatic external defibrillator (AED) on board a departing ambulance. However, the use of intravenous adrenaline is permitted only under direct medical instructions in limited locations. Paramedics are not legally allowed to declare death or terminate resuscitation attempts in the field unless the patients show obvious signs of death (e.g., corruption, rigor mortis) and medical physicians have confirmed the declaration via telephone. Therefore, most EMS-treated OHCA patients are transported to hospitals.

### Statistical analyses

Overall, no imputation method for missing data was used. The chi-squared test was used to compare categorical variables between groups with favorable and unfavorable neurological outcomes. Furthermore, since continuous variables were not normally distributed, the Mann–Whitney U test was performed to analyze continuous variables. Values are presented as median (interquartile range [IQR]) for continuous variables and as percentages for categorical variables. Multivariable logistic regression was conducted for selected variables related to favorable neurological outcomes. Variables including age, sex, witnessed arrest, bystander CPR, initial shockable pre-hospital rhythm, and total CPR duration were selected based on previous studies [2, 3, 16, 24]. There were no more extra variables added to the logistic regression model.

Model calibration was assessed using the Hosmer–Lemeshow goodness-of-fit test. As an estimate of effect size and variability, odds ratios (ORs) with 95% confidence intervals (CIs) were calculated. We stratified patients into four subgroups by combining important variables that showed a high association with favorable outcomes based on the multivariable logistic regression analysis results.

The dynamic probability and cumulative proportion of favorable neurological outcomes at discharge were calculated for all eligible participants stratified by significant variables. The dynamic probability of favorable neurological outcome at discharge < 1% in the selected group indicates the proportion of the favorable neurological outcome at discharge was less than 1% in all patients in the selected group. The cumulative proportion > 99% refers 99% of patients with favorable neurological outcomes were discharged in the selected group. We measured the optimal cut-off time of pre-hospital and total CPR duration, which is the time of CPR duration that has maximum sensitivity and specificity for favorable neurological outcomes at discharge. The shortest distance between each point on the receiver operating characteristic curve and the upper left corner was considered the optimal cut-off CPR duration for a favorable neurological outcome at discharge.

Analyses were performed using R-project version 3.6.2 (package “rms” version 5.1), SAS (version 9.4), and SPSS version 22.0 (IBM Corp., Armonk, NY, USA). Statistical significance was set at *P* < 0.05.

## Results

### Characteristics and pre-hospital and in-hospital resuscitation-related variables

**Fig. 1:**
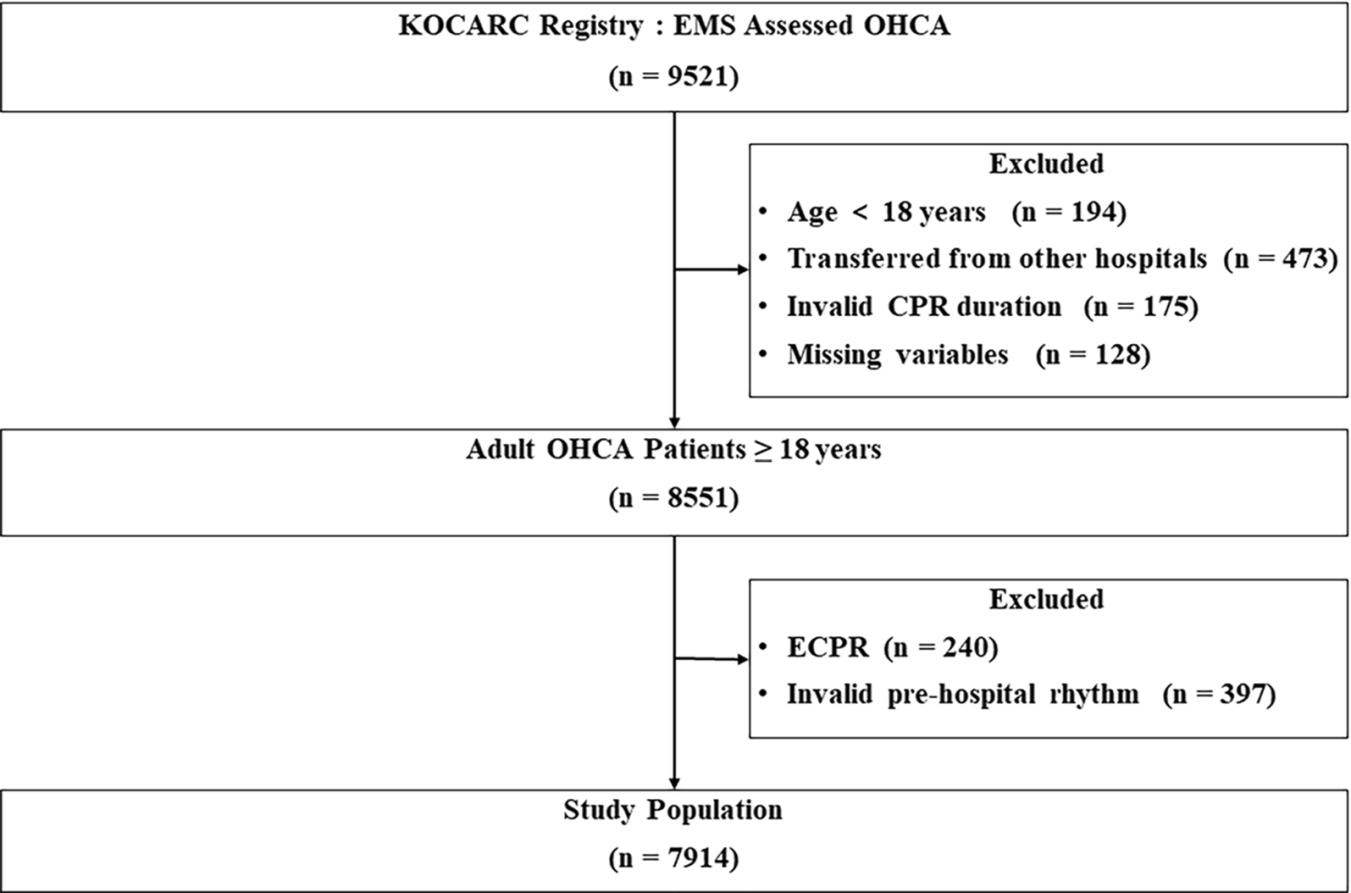
Flowchart of the study patient selection process and outcomes. CPR, cardiopulmonary resuscitation; ECPR, extracorporeal cardiopulmonary resuscitation; EMS, emergency medical service; OHCA, out-of-hospital cardiac arrest

Among the 7914 participants, 577 (7.3%) had favorable neurological outcomes (Fig. 1). Baseline characteristics, resuscitation-related variables, laboratory values, and treatments were compared between patients with favorable and unfavorable outcomes (Table 1). Patients with favorable outcomes were younger, were predominantly male, had a higher rate of witnessed arrest, had a higher provision of bystander CPR, and had a pre-hospital shockable rhythm. No intergroup differences were observed in the EMS response time (from EMS call to scene arrival) or transport time (from scene departure to hospital arrival). The unfavorable neurological outcomes group had longer scene times (from scene arrival to scene departure) with a higher frequency of pre-hospital adrenaline use and pre-hospital advanced airway trials. In-patient interventions, such as therapeutic hypothermia and coronary angiography, were performed more frequently in patients with favorable neurological outcomes. Serum cardiac markers and initial serum lactate levels were lower in these patients despite higher hemoglobin and pH levels.

**Table 1:** Characteristics of the favorable versus unfavorable outcome groups

	All patients (N = 7914)	Favorable outcome (n = 577)	Unfavorable outcome (n = 7337)	*P* value
**Baseline characteristics**				
Age (years)	71.0 (58.0–80.0)	56.0 (47.0–65.0)	73.0 (59.0–81.0)	< 0.001
Male sex, (%)	5116 (64.6)	452 (78.3)	4664 (63.6)	< 0.001
Underlying disease, n (%)				
Hypertension	3191 (40.3)	216 (37.4)	2975 (40.5)	0.14
Diabetes	2008 (25.4)	90 (15.6)	1918 (26.1)	< 0.001
Dyslipidemia	356 (4.5)	38 (6.6)	318 (4.3)	0.01
Arrest location, n (%)				< 0.001
Home	5062 (64.0)	221 (38.3)	4841 (66.0)	
Other	1893 (23.9)	252 (43.7)	1641 (22.4)	
Unknown	959 (12.1)	104 (18.0)	855 (11.7)	
**Event characteristics, n (%)**				
Witnessed arrest	4675 (59.1)	481 (83.4)	4194 (57.2)	< 0.001
Bystander CPR	4043 (51.1)	353 (61.2)	3690 (50.3)	< 0.001
Pre-hospital AED use	90 (1.1)	14 (2.4)	76 (1.0)	0.002
Initial documented arrest rhythm				< 0.001
VF/PVT	1467 (18.5)	469 (81.3)	998 (13.6)	
PEA	1783 (22.5)	76 (13.2)	1707 (23.3)	
Asystole	4640 (58.6)	31 (5.4)	4609 (62.8)	
Unknown	24 (0.3)	1 (0.2)	23 (0.3)	
**EMS intervention, n (%)**				
Advanced airway attempted	6340 (80.1)	401 (69.5)	5939 (80.9)	< 0.001
Adrenaline administered	1048 (13.2)	27 (4.7)	1021 (13.9)	< 0.001
Any defibrillation delivered	1927 (24.3)	491 (85.1)	1436 (19.6)	< 0.001
Pre-hospital ROSC	1063 (13.4)	498 (86.3)	565 (7.7)	< 0.001
**EMS time interval, min**				
Time from EMS call to scene arrival	7.0 (5.0–10.0)	7.0 (5.0–8.0)	7.0 (5.0–10.0)	0.62
Time from scene arrival to scene departure	12.0 (8.0–18.0)	10.0 (7.0–14.0)	13.0 (9.0–18.0)	< 0.001
Time from scene departure to hospital arrival	9.0 (6.0–13.0)	10.0 (7.0–14.0)	9.0 (6.0–13.0)	0.14
**Laboratory data**				
Hb, g/dL	11.9 (9.8–13.9)	14.4 (13.1–15.4)	11.6 (9.5–13.6)	< 0.001
CK-MB, ng/mL	4.3 (2.2–9.3)	2.8 (1.6–4.7)	4.6 (2.3–10.2)	< 0.001
Initial pH	6.9 (6.8–7.1)	7.3 (7.1–7.3)	6.9 (6.8–7.0)	< 0.001
Initial lactate, mmol/L	12.3 (9.0–15.0)	8.3 (6.0–11.4)	12.8 (9.6–15.0)	< 0.001
**In-patient intervention, n (%)**				
Therapeutic hypothermia	591 (7.5)	179 (32.3)	412 (6.8)	< 0.001
CAG	815 (10.3)	427 (76.4)	388 (6.3)	< 0.001
PCI	263 (3.3)	147 (26.7)	116 (1.9)	< 0.001
CABG	34 (0.4)	21 (3.8)	13 (0.2)	< 0.001
Pacemaker insertion	54 (0.7)	34 (6.2)	20 (0.3)	< 0.001
ICD insertion	69 (0.9)	64 (11.6)	5 (0.1)	< 0.001
TPA	196 (2.5)	82 (14.9)	114 (1.9)	< 0.001
**CPR duration, min**				
Pre-hospital CPR duration	19.0 (14.0–24.0)	7.0 (4.0–12.0)	19.0 (15.0–25.0)	< 0.001
In-hospital CPR duration	19.0 (9.0–29.3)	0.0 (0.0–0.0)	20.0 (11.0–30.0)	< 0.001
Total hospital CPR duration	40.0 (28.0–51.0)	8.0 (5.0–15.0)	41.0 (30.0–52.0)	< 0.001

All patient data are presented as reference values only. Data are shown as median (interquartile range) or number (%)

AED, automated external defibrillator; CABG, coronary artery bypass graft; CAG, coronary angiography; CPR, cardiopulmonary resuscitation; EMS, emergency medical service; ICD, implantable cardioverter defibrillator; PCI, percutaneous coronary intervention; PEA, pulseless electrical activity; PVT, pulseless ventricular tachycardia; ROSC, return of spontaneous circulation; TPA, tissue plasminogen activator; VF, ventricular fibrillation

The patients with unfavorable neurological outcomes at discharge had longer pre-hospital and total CPR durations (median, 19.0 min [IQR 15.0–25.0] and 41.0 min [IQR 30.0–52.0], respectively) than patients with favorable neurological outcomes (median, 7.0 min [IQR 4.0–12.0] and 8.0 min [IQR 5.0–15.0], respectively).

### Associated factors for favorable neurological outcomes at discharge

Table 2 shows the results of the multivariate logistic regression analysis of favorable neurological outcomes at discharge. An initial shockable rhythm (adjusted OR 10.7; 95% CI 8.0–13.9; *P* < 0.001) and witnessed arrest (adjusted OR 1.87; 95% CI 1.37–2.55; *P* < 0.001) were the main factors positively associated with favorable neurological outcomes at discharge. The total CPR duration (adjusted OR 0.89; 95% CI 0.88–0.90; *P* < 0.001) was inversely associated with a CPC of 1–2 at discharge. We stratified the patients into subgroups by combining the main associated factors (shockable rhythm + witnessed, non-shockable rhythm + witnessed, shockable + unwitnessed, and non-shockable + unwitnessed groups).

**Table 2:** Logistic regression analysis of survival with favorable outcomes

Variable	Crude OR (95% CI)	*P* value	Adjusted OR (95% CI)	*P* value
Age (years)	0.95 (0.95–0.96)	< 0.001	0.96 (0.95–0.97)	< 0.001
Male sex	2.07 (1.69–2.54)	< 0.001	1.07 (0.80–1.44)	0.65
Witnessed arrest (EMS or bystander)	3.75 (3.00–4.70)	< 0.001	1.87 (1.37–2.55)	< 0.001
Bystander CPR	1.56 (1.31–1.85)	< 0.001	0.99 (0.76–1.29)	0.95
Initial shockable rhythm	27.58 (22.15–34.36)	< 0.001	10.56 (8.00–13.92)	< 0.001
Total CPR duration, min	0.85 (0.84–0.86)	< 0.001	0.89 (0.88–0.90)	< 0.001

CI, confidence interval; CPR, cardiopulmonary resuscitation; EMS, emergency medical service; OR, odds ratio

### Dynamic probability of favorable neurological outcomes at discharge and cumulative proportion of total CPR duration

**Fig. 2:**
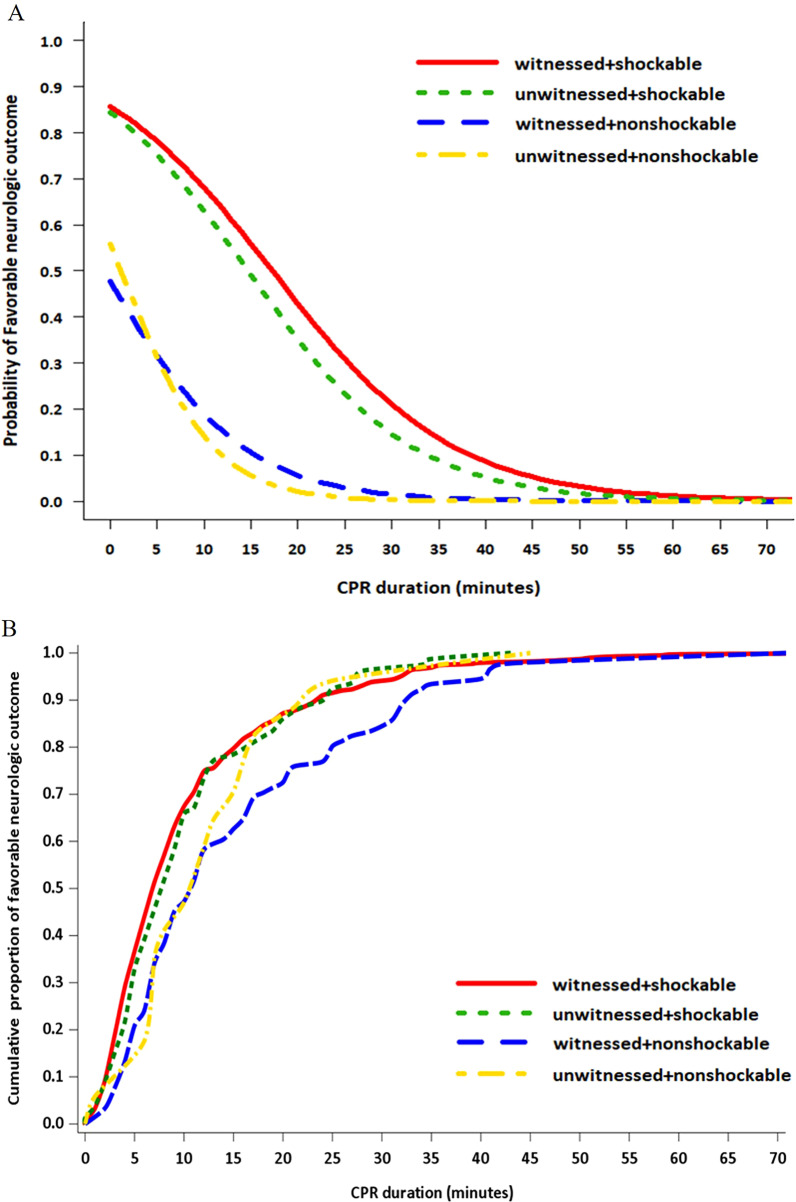
Dynamic probability (**A**) and cumulative proportion (**B**) of favorable neurological outcome at discharge according to total CPR duration. CPR, cardiopulmonary resuscitation

Figure 2 shows the dynamic probability and cumulative proportion of favorable neurological outcomes at discharge according to total CPR duration. After 30 min of CPR, the probability of favorable outcomes in the subgroups decreased in the following order: shockable rhythm + witnessed, shockable rhythm + unwitnessed, non-shockable rhythm + witnessed, and non-shockable rhythm + unwitnessed (21.0%, 14.6%, 1.5%, and 0.3%, respectively). After 40 min of CPR, the probabilities decreased to 8.6%, 5.1%, 0.4%, and 0.03%, respectively. For the same subgroups, the total CPR duration beyond which the dynamic probability of a favorable neurological outcome < 1% was 62 min, 55 min, 34 min, and 24 min, respectively (Fig. 2A, Additional file 1: Table S1), while the total CPR duration to achieve a > 99% cumulative proportion of a CPC of 1–2 at discharge was 53 min, 43 min, 71 min, and 45 min, respectively (Fig. 2B, Additional file 1: Table S1).

### Cut-off for pre-hospital and total cpr duration for favorable neurological outcomes at discharge

Table 3 shows the cut-off for pre-hospital and total CPR duration for a favorable neurological outcome at discharge. In all patients, the cut-off for pre-hospital CPR duration for a favorable neurological outcome at discharge was 12 min regardless of the initial rhythm. In addition, the cut-offs for total CPR duration for favorable neurological outcomes at discharge were 25 min for patients with initial shockable rhythms and 21 min for patients with non-shockable rhythms. When pre-hospital ROSC was achieved, the minimum cut-offs for the pre-hospital CPR duration were 10 and 12 min in patients with shockable and non-shockable rhythms, respectively. However, when pre-hospital ROSC was not achieved, the cut-offs for pre-hospital and total CPR duration were 14 and 36 min in patients with shockable rhythm and 12 and 26 min in patients with non-shockable rhythm, respectively.

**Table 3:** Optimal cut-off for pre-hospital and total CPR duration stratified by pre-hospital ROSC and initial shockable rhythm for favorable neurological outcomes at discharge

	Pre-hospital CPR duration				Total CPR duration			
	Cut-off time	Sensitivity	Specificity	AUC (95% CI)	Cut-off time	Sensitivity	Specificity	AUC (95% CI)
**Total population**								
Shockable	12 min	0.81	0.78	0.83 (0.81–0.85)	25 min	0.79	0.92	0.90 (0.89–0.92)
Non-shockable	12 min	0.85	0.76	0.83 (0.79–0.88)	21 min	0.91	0.78	0.91 (0.87–0.94)
**Pre-hospital** **ROSC (+)**								
Shockable	10 min	0.48	0.81	0.66 (0.62–0.71)	12 min	0.54	0.79	0.69 (0.64–0.73)
Non-shockable	12 min	0.78	0.80	0.83 (0.78–0.89)	17 min	0.73	0.83	0.85 (0.80–0.90)
**Pre-hospital** **ROSC (-)**								
Shockable	14 min	0.85	0.54	0.73 (0.64–0.82)	36 min	0.81	0.86	0.87 (0.81–0.94)
Non-shockable	12 min	0.85	0.71	0.80 (0.72–0.88)	26 min	0.85	0.73	0.86 (0.80–0.92)

AUC, area under the curve; CI, confidence interval; CPR, cardiopulmonary resuscitation; ROSC, return of spontaneous circulation

### Dynamic probability and cumulative proportion of favorable neurological outcomes at discharge by total CPR duration in patients without pre-hospital ROSC

**Fig. 3:**
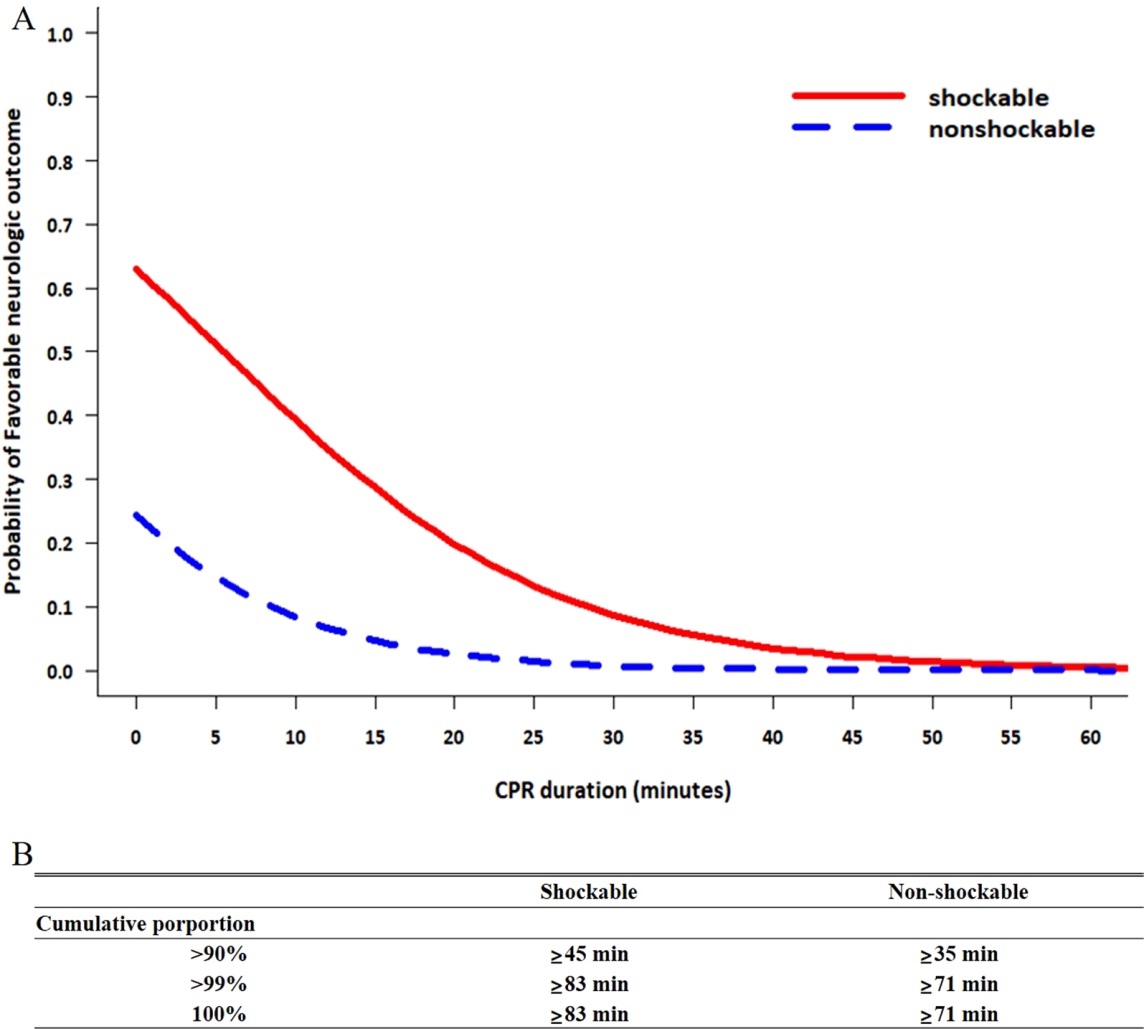
Dynamic probability (**A**) and cumulative proportion (**B**) of favorable neurological outcome at discharge in patients without pre-hospital ROSC. CPR, cardiopulmonary resuscitation; ROSC, return of spontaneous circulation

After 30 min of CPR, the probability of a favorable outcome according to the total CPR duration decreased to 8.7% and 0.7% in the shockable and non-shockable rhythm groups, respectively. The total CPR duration beyond which the dynamic probability of a favorable neurological outcome < 1% were 54 and 28 min, respectively, while the total CPR duration to achieve a > 99% cumulative proportion of a CPC of 1–2 at discharge were 83 min and 71 min, respectively (Fig. 3).

## Discussion

This multicenter retrospective observational study based on reliable pre-hospital and in-hospital variables showed that the upper limits of resuscitation times and optimal pre-hospital and total CPR durations differed among the stratified groups by major determinant factors. Similar to other studies [2, 3, 16, 24], an initial shockable rhythm and witnessed arrest were major factors determining favorable neurological outcomes. This study showed that males had a high likelihood of favorable neurological outcomes. However, there were some etiological differences according to sex. The males were younger and likely to have initial shockable rhythms (Additional file 2: Table S2). These differences made male patients seem to have a better prognosis.

In this study, the upper limit of total CPR duration in the shockable rhythm group (55–62 min) was longer than that in the non-shockable rhythm group (24–34 min), a finding that was similar to the results of Grunau et al. [24], who showed that the total CPR duration with a probability of favorable neurological outcomes < 1% were 48 and 15 min in the shockable and non-shockable rhythm groups, respectively. The reason for this is the difference in the characteristics of shockable and non-shockable rhythms. A shockable rhythm is caused by electrical changes due to myocardial ischemia, while a non-shockable rhythm is caused by poor perfusion and tissue hypoxia due to non-cardiac causes or spontaneous or electrical termination of shockable rhythm. Patients with an initial non-shockable rhythm may have more comorbidities and delayed initial recognition and response [25, 26]. Therefore, favorable neurological outcomes are more likely in patients with a shockable rhythm, even if prolonged CPR is required. Different CPR durations are recommended for different patient groups stratified by their characteristics.

Grunau et al. recommended that patients be transported to hospitals for ECPR within 8–24 min [27]. Similarly, we recommend 12 min as the optimal pre-hospital CPR duration cut-off, within which patients with OHCA should be transported to the hospital for evaluation of the cause of the OHCA and clinical decisions about ECPR implementation. Furthermore, this study showed that total CPR durations of 25 and 21 min are optimal for transitioning to ECPR and achieving favorable neurological outcomes in patients with shockable and non-shockable rhythms. Similarly, Kim et al. showed that the optimal cut-off for total conventional CPR is 21 min, beyond which ECPR should be considered [7]. Moreover, French medical scientific societies recommend ECPR in patients with refractory cardiac arrest with CPR performed for > 30 min [28]. ECPR implemented within 60 min results in favorable neurological outcomes [6, 7, 29]. In patients with an initial shockable rhythm with and without witnessed arrest, we revealed that total CPR for 62 min and 55 min, respectively, are the upper limits of conventional CPR for the dynamic probability of favorable neurological outcomes < 1%. This finding supports the hypothesis that implementing ECPR within 60 min of cardiac arrest in selected patients with an initial shockable rhythm may result in favorable neurological outcomes. In most cases, a shockable rhythm is caused by ischemic events that maintain myocardial viability and have a good response to ECPR. Although the effectiveness of ECPR in patients with a non-shockable rhythm is debatable, several studies have shown favorable neurological outcomes in patients with non-shockable rhythms [7–9]. We showed that the upper limit of total CPR duration in patients with an initial non-shockable rhythm with or without witnessed arrest are 34 and 24 min, respectively. The effect of ECPR is considered negligible after this time, which is similar to that observed in patients with an initial shockable rhythm. Although there are differences in the time interval from team activation to ECMO pump “ON” time due to regional variations in EMS systems, it usually takes > 20 min. Therefore, in patients with a non-shockable rhythm, ECPR should be considered quickly because they have a shorter time window for ECPR compared than those with a shockable rhythm.

Drennan et al. [30] disclosed that the application of the basic life support TOR rule (arrest not witnessed by EMS personnel, no ROSC, and no AED shock) at 20 min of resuscitation identified > 99% of survivors and favorable neurological outcomes. Similarly, we showed that the upper limit of total CPR duration for patients with an initial non-shockable rhythm with or without witnessed arrest were 34 and 24 min, respectively. Therefore, it might be feasible to apply the TOR rule within this time window to patients with a non-shockable rhythm in the field. However, the total CPR duration with a cumulative proportion of favorable neurological outcomes > 99% in patients with an initial non-shockable rhythm with and without witnessed arrest was 71 and 45 min, respectively. Thus, if prolonged CPR is performed, some patients with an initial non-shockable rhythm may have favorable neurological outcomes. Similarly, in patients with an initial shockable rhythm but without pre-hospital ROSC, the total CPR duration with a > 99% cumulative proportion of favorable neurological outcomes was 83 min, while the dynamic probability of < 1% favorable neurological outcomes was 54 min. Therefore, we recommend that treatment not be abandoned too early, even in patients with an initial non-shockable rhythm and in those without pre-hospital ROSC.

There have been many attempts to determine the upper limit of CPR duration beyond which resuscitation may be futile [1–4, 16, 17, 24]. Some studies focused on the upper limit of the pre-hospital CPR duration [1–3]. Even if few of these studies focused on total CPR duration, they excluded patients who underwent TOR in the field [4, 16, 24] and included patients who received bystander CPR only [17]. In conclusion, they determined the upper limit of CPR duration in limited patient groups. Conversely, this study focused on patients who did not experience TOR in the field and included all patients who underwent CPR by EMS at the pre-hospital level. Likewise, the present study could aid healthcare providers more clearly determine the appropriate duration of CPR performed at the pre-hospital level and the time to transfer from conventional CPR to ECPR in the hospital within the upper limit of CPR duration.

### Limitations

This study had some limitations. First, we included all OHCA patient data but did not consider the no-flow time that could affect favorable neurological outcomes. Second, CPR quality (compression rate, depth, and fraction rate) in pre-hospital and in-hospital settings can affect neurological outcomes; however, data in the KoCARC registry to estimate this are insufficient. Third, all patients, except those with traumatic OHCA, were enrolled in this study. Therefore, etiology may be a confounding factor affecting CPR duration and OHCA outcomes. Finally, the impact of witnessed arrest on the dynamic probability and cumulative proportion of favorable neurological outcomes was confirmed in all patients with OHCA but not in those without pre-hospital ROSC or an optimal cut-off time for favorable neurological outcomes. The OR of witnessed arrest (1.87) was less than that of the initial shockable rhythm (10.56). Since the impact of witnessed arrest on the dynamic probability and cumulative proportion of favorable neurological outcomes was small irrespective of the total CPR duration, this probably did not significantly impact the study results.

## Conclusions

The optimal cut-off for total CPR duration to achieve favorable neurological outcomes was 21–25 min. Thereafter, the effect of conventional CPR may decline, and the use of alternative CPR methods, such as ECPR, are recommended. Although the upper limits for CPR duration in patients with initial shockable and non-shockable rhythms are 55–62 min and 24–34 min, respectively, favorable neurological outcomes can be achieved with prolonged CPR according to each patient’s resuscitation-related factors.

## Supplementary Information

**Additional file 1: Table S1.** Probability of favorable neurological outcome (A) and cumulative proportion (B) of favorable neurological outcome at discharge by total CPR duration stratified by initial shockable rhythm and witnessed arrest

**Additional file 2: Table S2.** Comparison of characteristics by sex

## Data Availability

The data that support the findings of this study are available from the Korean Cardiac Arrest Research Consortium, but restrictions apply to the availability of these data, which were used under license for the current study, and so are not publicly available. Data are however available from the authors upon reasonable request and with permission of the Korean Cardiac Arrest Research Consortium.

## Abbreviations

AD: Advanced directives
AED: Automated external defibrillator
AUC: Area under the curve
CABG: Coronary artery bypass graft
CAG: Coronary angiography
CI: Confidence interval
CPC: Cerebral performance category
CPR: Cardiopulmonary resuscitation
ECPR: Extracorporeal cardiopulmonary resuscitation
ED: Emergency department
EMS: Emergency medical service
EMT: Emergency medical technician
ICD: Implantable cardioverter defibrillator
IQR: Interquartile range
KoCARC: Korean Cardiac Arrest Research Consortium
OHCA: Out-of-hospital cardiac arrest
OR: Odds ratio
PCI: Percutaneous coronary intervention
PEA: Pulseless electrical activity
PVT: Pulseless ventricular tachycardia
ROSC: Return of spontaneous circulation
TOR: Termination of resuscitation
TPA: Tissue plasminogen activator
VF: Ventricular fibrillation
