# Supplementary material for: Optimal cardiopulmonary resuscitation duration for favorable neurological outcomes after out-of-hospital cardiac arrest
Source: Scand J Trauma Resusc Emerg Med. 2022 Jan 15;30:5. doi: 10.1186/s13049-022-00993-8 (PMC8760684; doi:10.1186/s13049-022-00993-8)
Supplement: Supplementary file 1 — Additional file 1: Table S1. Probability of favorable neurological outcome (A) and cumulative proportion (B) of favorable neurological outcome at discharge by total CPR duration stratified by initial shockable rhythm and witnessed arrest [file 13049_2022_993_MOESM1_ESM.docx]

Supplementary Table 1. Probability of favorable neurological outcome (A) and cumulative proportion (B) of favorable neurological outcome at discharge by total CPR duration stratified by initial shockable rhythm and witnessed arrest

|  | Shockable  + witnessed | Shockable  + unwitnessed | Non-shockable  + witnessed | Non-shockable  + unwitnessed |
| --- | --- | --- | --- | --- |
| A. Probability of favorable neurological outcome | | | | |
| < 1% | 62 min | 55 min | 34 min | 24 min |
| B. Cumulative proportion of favorable neurologic outcome | | | | |
| ≥90% | 24 min | 25 min | 33 min | 25 min |
| ≥99% | 53 min | 43 min | 71 min | 45 min |
| ≥100% | 83 min | 43 min | 71 min | 45 min |

CPR, cardiopulmonary resuscitation
