# Supplementary material for: Optimal cardiopulmonary resuscitation duration for favorable neurological outcomes after out-of-hospital cardiac arrest
Source: Scand J Trauma Resusc Emerg Med. 2022 Jan 15;30:5. doi: 10.1186/s13049-022-00993-8 (PMC8760684; doi:10.1186/s13049-022-00993-8)
Supplement: Supplementary file 2 — Additional file 2: Table S2. Comparison of characteristics by sex [file 13049_2022_993_MOESM2_ESM.docx]

Supplementary Table 2. Comparison of characteristics by sex

|  | Male  (n = 5,116) | Female  (n = 2,798) | *P* value |
| --- | --- | --- | --- |
| Age (years) | 66.2 ± 15.0 | 72.5 ± 15.6 | <0.001 |
| Witnessed arrest | 3057 (59.8%) | 1618 (57.8%) | 0.099 |
| Bystander CPR | 2566 (50.2%) | 1477 (52.8%) | 0.025 |
| Initial shockable rhythm | 1176 (23.0%) | 291 (10.4%) | <0.001 |
| Pre-hospital CPR duration, min | 19.6 ± 10.1 | 19.6 ± 8.9 | 0.952 |
| In-hospital CPR duration, min | 20.9 ± 16.4 | 20.2 ± 15.2 | 0.064 |
| Total hospital CPR duration, min | 40.5±20.4 | 39.8±18.5 | 0.125 |
| Favorable neurological outcome | 452 (8.8%) | 125 (4.5%) | <0.001 |

CPR, cardiopulmonary resuscitation
